# The immunological role of ADAMs in the field of gastroenterological chronic inflammatory diseases and cancers: a review

**DOI:** 10.1038/s41388-022-02583-5

**Published:** 2022-12-26

**Authors:** Jun Arai, Yumi Otoyama, Hisako Nozawa, Naoya Kato, Hitoshi Yoshida

**Affiliations:** 1 Division of Gastroenterology, Department of Medicine, Showa University School of Medicine, Tokyo, Japan; 2 Department of Gastroenterology, Graduate School of Medicine, Chiba University, Chiba, Japan

**Keywords:** Immunosurveillance, Immunotherapy

## Abstract

Metalloproteinases cleave transmembrane proteins that play critical roles in inflammation and cancers. Metalloproteinases include a disintegrin and metalloprotease (ADAM), which we previously examined using a fluorescence assay system, and described their association with resistance to systemic therapy in cancer patients. There are also many reports on the relation between ADAM expression and the prognosis of patients with gastroenterological chronic inflammatory diseases and cancers. Inhibiting their immunomodulating activity in chronic inflammation restores innate immunity and potentially prevents the development of various cancers. Among the numerous critical immune system-related molecules, we focus on major histocompatibility complex class I polypeptide-related sequence A (MICA), MICB, intracellular adhesion molecule (ICAM)-1, TNF-α, IL-6 receptor (IL-6R), and Notch. This review summarizes our current understanding of the role of ADAMs in gastroenterological diseases with regard to the immune system. Several Food and Drug Administration (FDA)-approved inhibitors of ADAMs have been identified, and potential therapies for targeting ADAMs in the treatment of chronic inflammatory diseases and cancers are discussed. Some ongoing clinical trials for cancers targeting ADAMs are also introduced.

## Introduction

The World Health Organization predicts that cancer will be the leading cause of death and the greatest barrier to increased life expectancy worldwide in the 21st century [1]. Colorectal cancer (CRC), gastric cancer, and hepatocellular carcinoma (HCC) are common gastroenterological malignancies and the second, third, and fourth leading causes of cancer-related death, respectively [1]. The increased incidence of chronic inflammatory disorders, such as non-alcoholic steatohepatitis (NASH) associated with metabolic syndrome, diabetes mellitus, inflammatory bowel disease, and the aging of society impact the increased number of cancer patients [2].

The recent development of tyrosine kinase and immune checkpoint inhibitors has led to advances in cancer immunotherapy [3] and is expected to improve outcomes in various cancers. The immune system plays a vital role in recognizing malignant tissues and inhibiting their growth [4]. One of the hallmarks of aging is systemic low-grade chronic inflammation, a phenomenon known as “inflammaging” [5]. A previous report showed that in vitro aging of human fibroblasts by repeated passaging significantly increases the secretion of matrix metalloproteases (MMPs), proteases, growth factors, and other extracellular matrix-modifying proteins [6]. This age-related process appears to drive chronic inflammation by increasing systemic levels of interleukin (IL)-1, IL-6, IL-1α, IL-1β, IL-33, granulocyte-macrophage colony-stimulating factor, interferon γ, tumor necrosis factor (TNF), and C-reactive protein, all of which have been implicated in multiple morbidities and mortality in the elderly [4, 7, 8].

In a genome-wide association study of patients with chronic hepatitis C, we identified the major histocompatibility complex class I polypeptide-related sequence A (MICA) as a marker of HCC susceptibility [9]. MICA is a natural killer group D ligand, and its expression on the surface of infected or cancerous cells induces their elimination by natural killer (NK) cells. Accumulation of membrane-bound MICA (mMICA) enhances NK cell cytotoxicity against hepatoma cells [10], and mMICA cleavage releases soluble MICA (sMICA), which acts as an immunological decoy in the serum to prevent antitumor activity [11]. sMICA bound to NK cells in sera of patients disturbs their activity and down-regulates a natural killer group D receptor [12].

Cancer escape from immunosurveillance via mMICA shedding is mainly accomplished by a disintegrin and metalloproteases (ADAMs). Numerous studies over the past two decades have shown that ADAMs and MMPs cleave mMICA in several cancer cell lines [13]. Importantly, increased ADAM expression is associated with poor prognosis in patients with various cancers (Table 1). The ADAM structure consists of an N-terminus prodomain, a zinc-binding metalloprotease domain, a disintegrin domain, a cysteine-rich region, an epidermal growth factor (EGF)-like domain, a transmembrane region, and a cytoplasmic C-terminal end [14]. The metalloprotease and disintegrin domains play crucial roles as proteases.

**Table 1:** Relation between ADAM expression and prognosis in gastroenterological cancers.

	Cancer type	ADAM	Prognosis	*P*-value	Biomarker	Ref
1	PDAC	ADAM8	OS	0.048	None	[50] Valkovskaya N et al.
2	PDAC	ADAM9	OS	<0.05	None	[53] Grützmann R et al.
3	CRC	ADAM15	OS	0.03	None	[62] Toquet C et al.
4	CRC	none	OS, PFS	0.03, 0.002	MICA	[23] Arai J et al.
5	HCC	ADAM9	OS	0.00035	None	[20] Théret N et al.
6	HCC	ADAM17	OS	0.037	None	[20] Théret N et al.
7	HCC	ADAM17	OS	0.001	iNOS, Notch1	[37] Wang R et al.
8	HCC	ADAM9	RFS	0.033	None	[75] Xiang LY et al.
9	HCC	ADAM21	RFS, OS	0.001, 0.003	None	[87] Honda H et al.
10	Esophageal Ca	ADAM17	OS	<0.05	EGFR	[93] Liu HB et al.
11	Gastric Ca	ADAM17	OS	<0.05	None	[98] Shou ZX et al.
12	Gastric Ca	ADAM17	5 yr survival rate	<0.05	None	[99] Zhang TC et al.
13	Gastric Ca	ADAM17	DFS	0.038	None	[100] Aydin D et al.
14	Gastric Ca	ADAM17	OS	<0.001	None	[101] Fang W et al.
15	Gastric Ca	ADAM17	OS	<0.05	None	[102] Li W et al.

The impact of ADAM expression in gastroenterological cancers is shown in terms of prognosis and biomarker.

*PDAC* Pancreatic ductal adenocarcinoma, *CRC* colorectal carcinoma, *HCC* hepatocellular carcinoma, *iNOS* inducible nitric oxide synthase, *EGFR* epidermal growth factor receptor.

The most widely studied ADAMs are ADAM10 and ADAM17, which are very similar in structure and function [15]. ADAM17 is unique because the cysteine-rich and EGF-like domains are replaced by a membrane-proximal domain and a small stalk sequence termed Conserved ADAM seventeeN Dynamic Interaction Sequence (CANDIS). These two domains appear to be involved in substrate recognition and binding [16]. Furthermore, in ADAM17, the CANDIS region interacts with the cell membrane, thereby regulating its protease activity [17]. The processing and activation of ADAM17, like almost all ADAMs with MMP-like activity, are regulated at multiple levels [15].

This review summarizes our current understanding of the role of ADAMs in gastroenterological chronic inflammatory diseases and cancers. In particular, we focus on the vital role of ADAMs in escaping immunosurveillance.

## Proteolytic shedding

The role of ADAMs in disease pathophysiology is well characterized [14, 18, 19]. Among their domains, the metalloprotease and disintegrin domains are better characterized in terms of biological function. ADAMs are considered to be sheddases, since most of their substrates are membrane-bound precursors. Substrates include growth factors, chemokines, and adhesion molecules and their receptors. Sometimes, extracellular matrix components also act as substrates [20]. However, only 50% of ADAMs (ADAM8, 9, 10, 12, 15, 17, 19, 20, 21, 28, 30, and 33) are functional proteases, as indicated by the presence of the HEXGHXXGXXHD motif in their catalytic domain [14, 20].

The following sections describe the relationship between ADAMs and the development of gastroenterological cancers with regard to chronic inflammation. Among the numerous critical immune system-related molecules shown in Table 2, we focus on MICA, major histocompatibility complex class I polypeptide-related sequence B (MICB), intracellular adhesion molecule (ICAM)-1, TNF-α, IL-6 receptor (IL-6R), and Notch.

**Table 2:** Principal substrates of ADAMs in the field of gastroenterology.

ADAM	Other names	Substrates
ADAM9	MDC9, meltrin γ	ADAM10, EGF, HB-EGF, MICA, TNF-α
ADAM10	MADM	EGF, HB-EGF, IL-6R, MICA, Notch
ADAM17	TACE	EGF, HB-EGF, ICAM-1, IL-6R, MICA, MICB, Notch, TGF-α, TNF-α

The critical immune system-related molecules, which are principal substrates of ADAMs in the field of gastroenterology, are shown.

*MDC9* metalloproteinase/disintegrin/cysteine-rich 9, *MADM* Mammalian disintegrin-metalloprotease, *TACE* tumor necrosis factor-α converting enzyme.

## Specific molecules targeted by ADAMs

Previous studies investigated the substrates shed by active ADAM proteases and matrix metalloproteases [21]. In Section 3, we describe the molecular targets of ADAMs in inflammation and cancer.

### MICA/MICB

Among all NK receptor ligands, MICA and MICB are the most thoroughly investigated. As noted above, we identified MICA as a marker of HCC susceptibility [9]. In our previous investigation using human HCC cell lines, knockdowns of ADAM9, 10, and 17 by RNA interference reduced MICA shedding, while the knockdown of ADAM 17 also reduced MICB shedding [22]. In human CRC cell lines, ADAM10 and ADAM17 contributed to MICA shedding [23]. In chronic inflammation, the increased expression of mMICA with an inhibitor of MICA shedding enhanced NK cell-mediated cytotoxicity, thereby reinforcing immunosurveillance [10, 22].

### ICAM-1

Chen et al. reported that soluble ICAM-1 (sICAM-1) was associated with HCC incidence among patients with liver cirrhosis [24]. Potential mechanisms involve sICAM-1-mediated inhibition of interactions between tumor-specific T cells and cancer cells, and shedding by tumor cells, which promotes angiogenesis [25]. ICAM-1 is the main ligand for β2-integrins [26]. ICAM-1 expressed on endothelial cells in inflamed tissues binds to leukocytes and facilitates their transendothelial migration to the inflammation site. Although the expression of ICAM-1 in normal human liver cells is low, given its role in mediating leukocyte migration, ICAM-1 up-regulation has been associated with chronic inflammation and autoimmune liver diseases, and allergic diseases [27]. ICAM-1 is also expressed in many types of tumors and plays a critical role in tumor growth [28]. Of note, sICAM-1, another substrate of ADAM17, decreases in a dose-dependent manner in human HCC cells by lomofungin treatment [22]. These pleiotropic activities of lomofungin may result in anti-HCC effects. sICAM-1 levels are associated with tumor growth because it inhibits interactions between tumor-specific T cells and cancer cells. sICAM-1 shed by tumor cells also promotes angiogenesis. Therefore, inhibition of its shedding plays a vital role in reducing the tumor growth.

### TNF-α

Initially, TNF-α was considered to be pro-inflammatory. However, preclinical and clinical studies have shown that it also has anti-inflammatory and immunomodulatory effects [29]. ADAM17 was initially identified by its ability to release the soluble, inflammatory form of TNF-α from its precursor [30]. Thus, ADAM17 is a TNF-α converting enzyme and a metalloprotease. The antitumor immunity is suppressed by the influence of TNF-α on regulatory T cells via soluble TNF-α binding to the TNF receptor type 2 (TNFR2) [29]. Therefore, from the viewpoint of TNF-α, suppressing ADAM17 function is a promising strategy to strengthen antitumor immunity.

### IL-6R

Membrane-bound IL-6R and soluble IL-6R (sIL-6R) are present in high amounts in the serum of healthy individuals. They mediate the inflammatory response in all human cells [31]. IL-6 is a pleiotropic cytokine with a crucial role in immune and inflammatory reactions. In classic signaling, IL-6 binds to membrane-bound IL-6Rs, mainly expressed on hepatocytes and immune cells [32]. The association of the resulting complexes and consequent dimerization of gp130 initiates signaling through a signal transducer and activator of transcription-3 (STAT3). In trans-signaling, IL-6 binds to sIL-6Rs, produced via cleavage of membrane-bound IL-6Rs; IL-6/sIL-6 complexes stimulate cells that express gp130 but IL-6Rs [32, 33]. The cleavage of membrane-bound IL-6Rα and consequent release of sIL-6Rα by ADAM17 has been reported [31, 34]. In support of the role of IL-6 trans-signaling in inflammation, Yamaguchi et al. showed that blocking signaling with an anti-IL-6Rα antibody enhanced steatosis but reduced inflammation in mice with NASH [35].

IL-6 is a multifunctional nuclear factor κB-regulated cytokine that acts as a critical tumor promoter during early CRC tumorigenesis. The proliferative and survival effects of IL-6 are primarily mediated via STAT3. The nuclear factor κB/IL-6/STAT3 cascade is an important regulator of these effects in tumor-initiating intestinal epithelial cells [36].

### Notch

Notch signaling in cancer stem cells promotes cancer progression and requires Notch cleavage by ADAMs [37, 38]. Notch signaling pathways are composed of Notch receptors, their ligands, and DNA-binding proteins. To date, four transmembrane Notch receptors (Notch1–4) and two families of Notch ligands (Delta and Serrate) have been found in mammals [38].

The silencing of Notch1–4-known substrates of ADAMs [35, 36], via RNA interference attenuates Delta-like 4-mediated induction of nitric oxide synthase in human macrophages. Thus, Notch signaling via Delta-like 4 is critical for the pro-inflammatory activation of macrophages [39].

Importantly, in patients with HCC, increased tumor-associated macrophage (TAM) density is associated with large tumor size, advanced TNM stage, intrahepatic metastasis, disease recurrence, and poor overall survival (OS) [40].

In hepatitis B virus-related HCC, higher expression of Notch3 is associated with poor OS (*P* = 0.002) and shorter recurrence-free survival (RFS) (*P* = 0.001) [41]. Overexpression of Notch1 and Notch4 are prognostic markers in patients with HCC after curative resection, indicating shorter disease-free survival (Notch1, *P* = 0.029) and shorter disease-specific survival (Notch1 and Notch4, *P* = 0.039 and 0.012, respectively) [42].

In the intestine, Notch is essential for the polarization of inflammatory and regenerative macrophages and functional differentiation of dendritic cell populations [43]. ADAM10 is rate-limiting for Notch activation in crypt homeostasis [44, 45]. Increased Notch activity is significantly associated with decreased goblet cell differentiation in patients with ulcerative colitis [46, 47]. Although assessing ADAM10 function in intestinal homeostasis is technically challenging, these observations suggest that ADAM10 is an important and possibly a rate-limiting regulator of Notch signaling during intestinal inflammation and development of colitis-associated cancer [48]. The first-in-human phase I study of an oral Notch inhibitor in patients with advanced cancer showed that while the drug was well tolerated with only gastrointestinal side effects, the efficacy was suboptimal [49]. Future studies should explore oral Notch inhibitors on an intermittent schedule combined with either chemotherapy or targeted agents to achieve greater clinical efficacy.

## Functions of ADAMs in the field of gastroenterology

There are many reports on the relation between ADAM expression and the prognosis of patients with gastrointestinal diseases (Table 1).

### Pancreatic ductal adenocarcinoma (PDAC)

Aberrant expressions of ADAM8, 9, 15 and 17 have been observed in PDACs, and overexpression of these proteases increases the invasiveness and aggressiveness of PDACs [50–53]. ADAM8 mRNA was significantly overexpressed in PDAC specimens when compared with that in normal pancreatic tissue (*P* = 0.0008). High ADAM8 mRNA and protein levels correlated with reduced survival time (*P* = 0.048 and 0.065, respectively) [50]. In that study, ADAM8 silencing inhibited the invasion, but not the growth of PDAC cells. The involvement of extracellular signal-regulated kinase 1/2 and MMPs in ADAM8-induced migration and invasiveness in PDAC cells has been reported, leading to high expression levels of ADAM8 correlated with poor clinical outcome [54]. It also revealed a peptidomimetic ADAM8 inhibitor, which prevents ADAM8 multimerization and affects ADAM8 function, thereby leading to reduced invasiveness. Additionally, its application in mice decreased tumor burden and metastasis of implanted pancreatic tumor cells. Thus, these data validate that ADAM8 is a promising target for PDAC therapy.

In a study by Oria et al., ADAM9 was prominently expressed in PDAC cells in a tissue microarray and increased ADAM9 expression correlated with advanced tumor grade (*P* = 0.027) and vasculature invasion (*P* = 0.017) [55]. In that study, silencing of ADAM9 in PDAC cells suppressed angiogenesis, cell migration, adhesion to different extracellular matrices, and anchorage-independent growth, suggesting that ADAM9 plays a key role in PDAC progression [55].

### Inflammatory bowel diseases

In patients with inflammatory bowel diseases such as ulcerative colitis, the risk of CRC development is much higher than in the general population [36]. Mosnier et al. reported that ADAM15 expression was higher in the epithelial cells of patients with inflammatory bowel disease than in individuals with a normal colon [56]. Moreover, ADAM15-positive epithelial cells were in close contact with α5β1-integrin-positive leukocytes in the crypt abscesses, and α5β1- and αvβ3-positive pericryptic myofibroblasts in the regenerative areas. Analysis of colonic biopsies revealed ubiquitous expression of active ADAM17 in a normal colon, but an increased expression in colons from patients with ulcerative colitis [57]. ADAM17 activity was also present in the mucosa of patients with Crohn’s disease, but there was no significant increase when compared with that in the control groups [57]. The biological therapy with anti-TNF-α monoclonal antibodies results in an effective response in only 60–70% of patients with Crohn’s disease.

Interestingly, down-regulation of ADAM17 mRNA was detected in inflamed and non-inflamed tissues of refractory patients who had received anti-TNF- α therapy [58]. Moreover, the role of anti-TNF-α monoclonal antibodies in antibody-dependent cellular cytotoxicity was indicated in studies using modified cell cultures, stably expressing membrane-bound TNF-α. As previously noted, ADAM17 cleaves membrane-bound TNF-α. Besides controlling the balance between soluble TNF-α and membrane-bound TNF-α, ADAM17 is important for the activity of FcγRIII. ADAM17 also cleaves membrane-bound FcγRIII receptors expressed on the surface of NK cells [58]. These two functions contribute to the escape from anticancer immunity. Therefore, ADAM17 is also a well-known therapeutic target to improve the immune response.

### CRC

Numerous studies have shown that ADAMs are upregulated in CRC tissue when compared with that in adjacent non-tumoral tissue. Since extracellular matrix components in the cancer stroma are produced by fibroblasts within cancer tissue, cancer-associated fibroblasts (CAFs) attribute to a desmoplastic reaction in CRC [59]. Desmoplastic reaction refers to the overgrowth of fibrous connective tissues around carcinoma cell nests. The degree of this overgrowth at the invasive front of CRC was previously reported as a promising prognostic indicator [60]. When CRC organoids were cultured with CAFs in extracellular matrix-coated plates, the expression of various ADAMs in CAFs was increased [59]. In particular, ADAM9 secreted by CAFs induces the shedding of heparin-binding epidermal growth factor on CRC cells, thereby enhancing the proliferation of CRC cells. ADAM9 also degrades the basement membrane extracellular matrix components, such as laminin and fibronectin, to promote migration and invasion. Consequently, ADAM9 expression was associated with a morphological desmoplastic reaction, thereby affecting cancer malignancy via tumor proliferation in CRC.

Blanchot-Jossic et al. reported that ADAM17 is overexpressed in colon carcinomas, regardless of the tumor stage, differentiation status, and EGF receptor (EGFR) expression [61]. Moreover, co-expression of ADAM17 and activated EGFRs, while not always proportional, suggests a role for ADAM17 in colon carcinoma growth and angiogenesis. Interestingly, in a study by Toquet et al., ADAM15 expression was significantly downregulated in poorly differentiated colon carcinomas, and ADAM15-positive patients had a shorter OS than ADAM15-negative patients (*P* = 0.03) [62].

ADAM10 has been shown to induce CRC metastasis via cleavage of the extracellular domain of cell adhesion molecule L1 (L1-CAM), a novel molecular target in β-catenin/T-cell factor signaling [63]. Overexpression of ADAM10 in CRC cells enhanced L1-CAM cleavage in vitro, and LI-CAM and ADAM10 expression in nude mice induced liver metastasis [63].

**Fig. 1:**
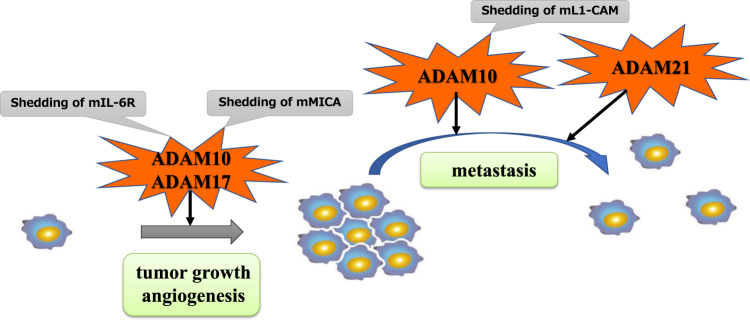
The mechanism of how ADAMs are associated with tumor progression in CRC. CRC cells advance as ADAM10 and ADAM17 cleave various membrane-bound molecules during growth, angiogenesis, and metastasis. mIL-6R: membrane-bound IL-6 receptor, mMICA: membrane-bound MICA, mL1-CAM: membrane-bound cell adhesion molecule L1.

In a study of 164 patients with CRC, the concentration of serum IL-6, the ligand to IL-6R cleaved by ADAM17 [31, 34], was associated with CRC progression and, therefore, may be a useful tumor marker to monitor the treatment course; nevertheless, serum IL-6 was not an effective independent prognostic indicator in that study [64]. However, prognostic significance was observed when an IL-6 threshold was used by Belluco et al. in their multivariate analysis. An IL-6 level of >10 pg/mL was a negative prognostic indicator of survival (relative risk = 1.820, *P* = 0.020) [65]. Moreover, patients with CRC with higher IL-6 concentrations (>10 pg/mL) had significantly lower 5-year survival rates than those with lower IL-6 concentrations (≤10 pg/mL) (*P* = 0.001) [65]. Additionally, the cleavage of membrane-bound IL-6Rs by ADAM17 has been reported [66]. As demonstrated in Fig. 1, CRC cells advance as ADAM10 and ADAM17 cleave mMICA or membrane-bound IL-6R in the tumor microenvironment, and furthermore, the shedding of membrane-bound L1-CAM influences metastasis.

### Chronic hepatitis

In severe alcoholic hepatitis (SAH), ADAM17 is involved in shedding, i.e., the cleavage and release of the soluble ectodomain of many membrane-bound pro-proteins including pro-TNF-α and CD163 [67, 68]. Maras et al. reported that the serum CD163 and TNF-α levels were significantly higher in patients with SAH who did not survive than in those who survived [68]. Increased CD163 expression on macrophages in inflammatory conditions, including SAH, has been reported [69].

NASH is emerging as a leading cause of chronic liver disease. Human genetic studies have shown that hypomorphic variations in the gene encoding c-mer tyrosine kinase (MERTK) protect against liver fibrosis [70]. Of note, in a genome-wide association study and several independent replication cohorts of patients with NASH or hepatitis C virus-related chronic hepatitis, naturally occurring variations in MERTK contributed to the development and progression of liver fibrosis [71, 72]. MERTK is a receptor mainly found on macrophages and is cleaved by ADAM17 [73]. Studies using NASH mouse models have shown that ADAM17-mediated MERTK cleavage in liver macrophages decreases during the steatosis to NASH transition and MERTK activation promotes, whereas its inactivation suppresses, liver fibrosis [70].

In a multivariate analysis, MICA alleles were associated with a high risk of histologic NASH and a low risk of focal hepatocyte necrosis and advanced fibrosis [74]. However, since the serum MICA concentration was not measured, whether MICA is a predictive biomarker for NASH remains unclear.

### HCC

**Fig. 2:**
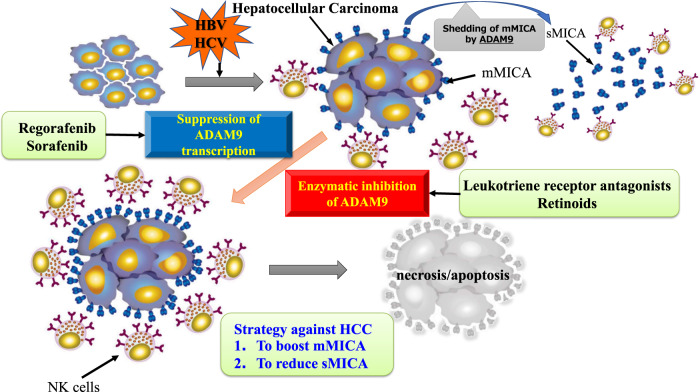
Combination therapy of ADAM9 inhibitors via two pathways could induce stronger NK cell-mediated cytotoxicity against HCC. In patients with chronic viral hepatitis, mMICA is expressed on the surface of inflammatory hepatocytes, which is an important trigger for NK cells to eliminate them. ADAM9 cleaves mMICA to escape from NK-mediated immunosurveillance, resulting in the development of HCC. The combination of agents targeting ADAM9 activity (leukotriene receptor antagonists and retinoids) and conventional multi-kinase inhibitors (sorafenib and regorafenib) represents a practical future therapeutic strategy to enhance the efficacy of cancer management and treatment. mMICA: membrane-bound MICA, sMICA: soluble MICA.

Increased expression of several ADAM family members, including ADAM8, 9, 10, 12, and 17, has been associated with HCC progression [20]. According to RNAseq data generated by The Cancer Genome Atlas Program Research Network, ADAM9, 10, and 17 are highly overexpressed in HCC tissue, indicating a worse prognosis [20]. The mRNA level of ADAM9 in HCC tissue is an independent prognostic factor for shorter RFS survival in hepatitis B virus-related HCC [75]. In terms of substrates for ADAM9, MICA has substantial functions in tumor immunology, especially targeted by NK cells [76]. In cohorts of patients with chronic hepatitis C, higher sMICA levels after viral eradication were associated with HCC progression to escape NK-mediated immunosurveillance [77]. The combination of agents targeting ADAM9 activity and conventional multi-kinase inhibitors represents a useful future therapeutic strategy to enhance the efficacy of cancer management and treatment (Fig. 2) [76, 78, 79].

Inducible nitric oxide synthase (iNOS) expression is also associated with HCC aggressiveness. iNOS and nitric oxide promote ADAM17-mediated Notch1 activation in liver cancer stem cells, leading to a more aggressive cancer phenotype [37]. In patients with HCC, the expression of the stem cell markers CD24 and CD133 correlates with greater iNOS expression and worse outcomes, and higher ADAM17 expression and Notch1 activation correlate with poor prognosis [37]. ICAM-1 was reported as a substrate for ADAM17 in human HCC [22]. Among a prospective cohort study to measure the baseline serum cytokines and other markers in 282 patients with both viral or non-viral chronic liver disease, sICAM-1 was associated with HCC development when multivariate analysis was applied (hazard ratio = 2.75, *P* = 0.041) [24]. Using another analysis with prospectively collected clinicopathological data of 36 patients with HCC after successful hepatectomy, higher sICAM‑1 was predictive of a worse OS and a lower RFS [80]. Furthermore, a single nucleotide polymorphism of another substrate for ADAM17, transforming growth factor-α (TGF-α), was reported to be an important factor in immunity, progression of inflammatory process, and carcinogenesis in patients with chronic hepatitis B virus infection [81]. Although the TGF-α expression level was not measured in each individual, TGF-α polymorphism was associated with hepatitis B virus clearance and HCC risk. Considering the molecular basis of hepatitis B virus-related HCC, Notch expression was upregulated in HCC when compared with that in paired peritumoral tissues. The hepatitis B virus X protein is thought to cause Notch overexpression. Furthermore, increased Notch3 expression was closely associated with the vascular invasiveness of HCC [82]. In addition, Yu et al. checked the single nucleotide polymorphisms of Notch pathway receptors in 465 patients with hepatitis B virus-related HCC who underwent surgery. They could confirm that single nucleotide polymorphisms of Notch pathway receptors were associated prognosis. Moreover, higher expression of Notch3 was associated with poor OS (HR = 2.11, *P* = 0.002) and shorter recurrence time of hepatitis B virus-related HCC (HR = 1.96, *P* = 0.001) [41].

CAFs are associated with HCC growth, metastasis, and intravasation [83]. CAFs secrete high levels of IL-6, and in response, HCC cells acquire stem cell-like characteristics. Notch is subsequently activated, and STAT3 is phosphorylated. Notch1 silencing abolishes the pro-tumorigenic effect of CAFs on HCC progression [84]. More importantly, Notch signaling can promote the progression of liver fibrosis by inducing the polarization of inflammatory and regenerative macrophages and activating hepatic stellate cells [85]. Considering the cytokines related to HCC, Myojin et al. confirmed that the progression-free survival and OS of the sIL-6-high group were significantly shorter than those of the sIL-6-low group [86]. However, these studies have not excluded the possibility that expression of HCC specimens is related to sIL-6 level; therefore, further research is needed.

In an analysis of HCC clinical specimens, ADAM21 positivity was associated with vascular invasion, large tumor size, high histological grade, and low OS and RFS, and in a multivariate analysis, it was an independent risk factor for OS (hazard ratio = 2.778, *P* = 0.003) and RFS (hazard ratio = 2.473, *P* = 0.001) [87]. These results suggest that ADAM21 plays a role in HCC metastasis and can serve as a prognostic marker for disease progression.

### Esophageal cancer

CAFs worsen survival by conferring therapy resistance to CRCs and esophageal cancers [88–90]. Examination of patient-derived CAFs identified IL-6 as the stromal driver of therapy resistance in esophageal cancer as revealed by the two following observations [91]. First, IL-6 activated the epithelial-to-mesenchymal transition in esophageal cancer cells, which enhanced treatment resistance, migratory capacity, and clonogenicity. Second, inhibition of IL-6 expression restored drug sensitivity in patient-derived organoid cultures. In addition, the analysis of gene expression profiles of patients with esophageal cancer identified ADAM12 as a noninflammatory-related serum marker for IL-6-producing CAFs, and serum levels of ADAM12 predicted unfavorable responses to neoadjuvant chemoradiation. Another recent investigation with a total of 140 specimens of esophageal cancer clarified that CAFs regulate immunosuppressive tumor-infiltrating lymphocyte populations in the tumor microenvironment via IL-6. In the investigation for CAFs and CD8+ or forkhead box protein 3 tumor-infiltrating lymphocytes by IHC, it was revealed that IL-6 blockade, or targeting CAFs, may improve preexisting tumor immunity and enhance the efficacy of conventional immunotherapies [92]. However, there is no evidence of the detailed description between ADAM expression and sIL-6 level.

In an analysis of 80 cases of esophageal squamous cell carcinoma, ADAM17 expression was directly associated with lymph node metastasis and the TNM stage (both *P* values <0.05) [93]. Moreover, ADAM17 and EGFR expression had prognostic significance using multivariate survival analysis (both *P* values <0.05). In addition, catalytically inactive members of the rhomboid family of proteases, iRhom2, are known to mediate the intracellular transport and maturation of ADAM17. Expression of iRhom2 lacking the extended amino-terminal cytoplasmic domain increases ADAM17 activity, TNFR shedding, and resistance to TNF-induced cell death. Cells from patients with a dominantly inherited cancer susceptibility syndrome called tylosis with esophageal cancer show amino-terminal mutations in iRhom2 [94]. Importantly, this result demonstrates that the loss of the amino terminus in iRhom2 impairs TNF signaling, despite enhancing ADAM17 activity. This could explain how mutations in the amino-terminal region contribute to the cancer predisposition syndrome, tylosis with esophageal cancer.

Owing to its role as a stress-induced transcriptional repressor of plasminogen activator inhibitor-1 expression, ADAM9 may promote tumor vascularization [95]. Its expression is associated with poor clinical outcomes in patients with esophageal cancer. In patients with early-stage (stage I and II) esophageal cancer who received curative esophagectomy, those with positive ADAM9 staining had a shorter survival time than those with negative ADAM9 staining (*P* < 0.01) [95].

### Gastric cancer

Several analyses have investigated the relationship between ADAM17 expression and the prognosis of patients with gastric cancer. In a meta-analysis of seven studies with 1757 patients, high ADAM17 expression correlated with poor prognosis [96]. In a study of 374 patients with gastric cancer, ADAM17 expression was significantly upregulated at both, the transcriptional and translational levels in gastric cancer tissue, but did not significantly correlate with patient survival [97].

The following studies, however, support an association between ADAM17 expression and gastric cancer prognosis.

1. Shou et al. reported that patients with gastric cancer with high ADAM17 expression had a longer mean survival time than patients with low expression (*P* < 0.05), particularly those with stage II gastric cancer (*P* < 0.01) [98]. Moreover, ADAM17 expression was an independent prognostic factor in multivariate survival analysis.
2. In 220 patients with stage I or II gastric cancer, the 5-year survival rate was significantly higher in the absence vs. presence of ADAM17 expression (both *P* values < 0.05); there was no correlation at stage III or IV [99].
3. Aydin et al. reported a higher recurrence rate (*P* = 0.032) and, consequently, a shorter median disease-free survival time (*P* = 0.004) in patients with gastric cancer with high vs. low ADAM17 expression [100]. They also identified ADAM17 as an independent prognostic factor for disease-free survival. ADAM17 expression correlated with (*P* = 0.019), but did not independently predict OS.
4. In the study by Fang et al., overexpression of ADAM17 in gastric cancer correlated with shorter survival times (*P* < 0.001) and was an independent prognostic indicator in a Cox proportional hazards model [101].
5. In a multivariate analysis conducted by Li et al., ADAM17 expression independently predicted significantly shorter survival times in patients with gastric cancer (*P* < 0.05), whereas ADAM9 expression did not [102]. Although most patients in this study expressed ADAM9, demonstrating the influence of ADAM9 on patient’s survival was challenging.

*Helicobacter pylori* (*H. pylori*) is the main risk factor for gastric cancer, with almost 90% of new cases of non-cardia gastric cancer attributed to this bacterium and class I carcinogen [103]. As described and portrayed in a recent study, *H. pylori* secretes proteases and transcriptionally upregulates a wide range of host ADAMs and MMPs, all of which can directly shed cytokines, promote the epithelial-mesenchymal transition, and disrupt lateral junction complexes [104]. In advanced stages of *H. pylori* pathogenesis, these proteases have been implicated in proliferation, tumor cell migration, invasive growth, angiogenesis, and processes related to the epithelial-mesenchymal transition. *H. pylori* infection disrupts the balance between gastric epithelial cell proliferation and apoptosis, which likely lowers the threshold for the development of gastric cancer [105].

**Fig. 3:**
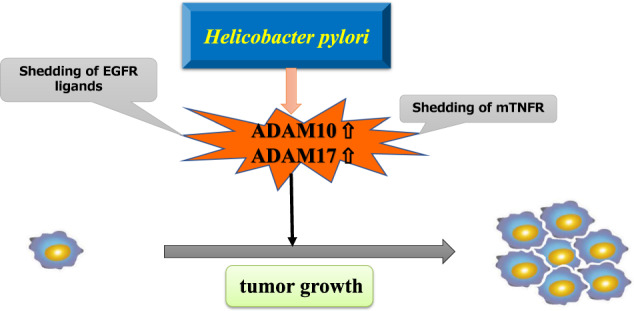
The mechanism of how ADAMs are associated with tumor progression in gastric cancer. H. pylori-induced chronic inflammation upregulates ADAM10 and ADAM17 expression, thereby increasing shedding of mTNFRs or EGFR ligands, and is also associated with tumor proliferation. EGFR epidermal growth factor receptor, mTNFR membrane-bound tumor necrosis factor receptor.

The cellular mechanisms by which *H. pylori* induces apoptosis include the activation of cytokine receptors, such as TNFRs [106]. ADAM17 cleaves TNFR1 and TNFR2 [107], and cleaved and soluble TNFRs (sTNFRs) levels are significantly higher in *H. pylori*-infected vs. uninfected patients. The combination of anti-sTNFR1 and anti-sTNFR2 monoclonal antibodies significantly increases TNF-induced cytotoxicity and apoptosis of gastric epithelial cells [106]. This result suggests that *H. pylori* protects gastric epithelial cells from TNF-induced apoptosis via sTNFRs production. Since the EGFR transactivation requires ADAM10, rather than ADAM17, for shedding the ectodomain of EGFR ligands, IL-8 dose-dependently released the EGFR ligands in human gastric cancer cells in vitro [108]. As shown in Fig. 3, *H. pylori*-induced chronic inflammation upregulates ADAM10 and ADAM17 expression, resulting in increased shedding of membrane-bound TNFRs or EGFR ligands, and is related to tumor proliferation and lower patient survival rates.

## ADAMs as immunotherapeutic targets

Metalloproteinases, including ADAM produced by cancer cells, are important therapeutic targets to prevent tumor invasion and metastasis. Although the pharmaceutical industry produced several orally active broad inhibitors, most of them were limited to phase I and phase II trials due to side effects including hepatotoxicity or musculoskeletal injury [109, 110]. Recently, Saha et al. reported that targeting the metalloprotease domains of ADAM proteases, particularly of ADAM10 and ADAM17, for the treatment of various diseases, has been unsuccessful so far mostly because the proteinase active site is highly conserved, resulting in toxicity due to the interactions of drugs with other matrix metalloproteases [111]. One potential solution is to target a specific ADAM. As summarized in Table 3, two approaches can be used to disrupt specific ADAM signaling during cancer treatment: the reduction of ADAM expression and the inhibition of ADAM activity. ADAM9 expression, for example, is transcriptionally and translationally repressed by tyrosine kinase inhibitors such as, sorafenib and regorafenib [76, 112]. Similarly, epirubicin represses ADAM10 transcription, thereby enhancing NK cell cytotoxicity against HCC cells [113]. Agents that inhibit ADAM9 activity include, leukotriene receptor antagonists [78] and retinoids [79]. Using a fluorescence assay system and diverse drugs approved by the Food and Drug Administration (FDA), we identified disulfiram and lomofungin as efficient enzymatic inhibitors of ADAM10 [114] and ADAM17 [22], respectively. The combination of regorafenib and an ADAM9 enzymatic inhibitor strongly inhibited MICA shedding than either of the agents alone [78], and the consequent accumulation of mMICA augmented NK cell cytotoxicity against human HCC cells [10]. The approach to explore beneficial medicines among the drugs approved by the FDA could provide us with a shortcut to develop new therapeutic strategies.

**Table 3:** Potential drugs targeting ADAM proteases.

	Cancer type	ADAM	Drugs	Biomarker	Ref
1	HCC	ADAM9(mRNA,protein)	sorafenib	MICA	[112] Kohga K et al.
2	HCC	ADAM9,ADAM10(mRNA,protein)	regorafenib	MICA	[76] Arai J et al.
3	HCC	ADAM9(enzyme)	LTRAs	MICA	[78] Arai J et al.
4	HCC	ADAM9(enzyme)	retinoids	MICA	[79] Otoyama Y et al.
5	HCC	ADAM10(mRNA)	epirubicin	MICA	[113] Kohga K et al.
6	HCC	ADAM10(enzyme)	disulfiram	MICA	[114] Goto K et al.
7	HCC	ADAM17(enzyme)	lomofungin	MICA, MICB, ICAM-1	[22] Arai J et al.
8	CRC	None	regorafenib	MICA	[23] Arai J et al.

Potential drugs targeting ADAM proteases in two approaches with gastroenterological cancers, (1) reduction of ADAM expression in transcriptionally and translationally and (2) inhibition of ADAM enzymatic activity, are shown.

*LTRAs* leukotriene receptor antagonists.

As determined by a literature search of PubMed and ClinicalTrials.gov, two clinical trials targeting ADAMs are ongoing as of August 2022. The first is a first-in-human study of IMGC936 in patients with advanced solid tumors (NCT04622774) [115]. IMGC936 is a novel antibody–drug conjugate targeted against ADAM9 and comprises a high-affinity humanized antibody site. As preclinical data, Scribner et al. reported that IMGC936 exhibits cytotoxicity in ADAM9-positive human tumor cell lines, along with bystander killing and potent antitumor activity in xenografts derived from tumors and human tumor cell lines [115]. A clinical trial combining an antibody-drug conjugate and immunotherapy is near at hand.

The second ongoing clinical trial (the Blood-borne Assessment of Stromal Activation in Esophageal Adenocarcinoma to Guide Tocilizumab Therapy) includes patients with esophageal cancers [116]. Its primary objective is to determine whether targeting stroma by tocilizumab in patients with highly activated stroma increases the efficacy of chemoradiotherapy. Patients will be grouped according to their expression level of ADAM12, which is a non-invasive blood-borne marker of stromal activation.

## Conclusion

This review summarized our current understanding of the immunomodulatory role of ADAMs in chronic inflammatory diseases and cancer in the field of gastroenterology.

## Data Availability

All the data are available under reasonable request. Material requests should be address to araiguma10@med.showa-u.ac.jp.
